# Developing a Feline Immunodeficiency Virus Subtype B Vaccine Prototype Using a Recombinant MVA Vector

**DOI:** 10.3390/vaccines10101717

**Published:** 2022-10-14

**Authors:** Luis A. F. Andrade, Alice F. Versiani, Edel F. Barbosa-Stancioli, Jenner K. P. dos Reis, Jordana Grazziela A. C. dos Reis, Flavio G. da Fonseca

**Affiliations:** 1 Laboratory of Basic and Applied Virology, Federal University of Minas Gerais, Belo Horizonte 31270-901, Brazil; 2 Vaccine Technology Center, Federal University of Minas Gerais (UFMG), Belo Horizonte 31270-901, Brazil; 3 Department of Pathology, University of Texas Medical Branch, Galveston, TX 77555-0132, USA; 4 Retroviruses Laboratory, Retrolab Federal University of Minas Gerais, Belo Horizonte 31270-901, Brazil

**Keywords:** feline immunodeficiency virus, modified vaccinia virus Ankara, vaccine

## Abstract

The *feline immunodeficiency virus* (FIV) is a retrovirus with global impact and distribution, affecting both domestic and wild cats. This virus can cause severe and progressive immunosuppression culminating in the death of felids. Since the discovery of FIV, only one vaccine has been commercially available. This vaccine has proven efficiency against FIV subtypes A and D, whereas subtype B (FIV-B), found in multiple continents, is not currently preventable by vaccination. We, therefore, developed and evaluated a vaccine prototype against FIV-B using the recombinant viral vector modified vaccinia virus Ankara (MVA) expressing the variable region V1–V3 of the FIV-B envelope protein. We conducted preclinical tests in immunized mice (C57BL/6) using a prime-boost protocol with a 21 day interval and evaluated cellular and humoral responses as well the vaccine viability after lyophilization and storage. The animals immunized with the recombinant MVA/FIV virus developed specific splenocyte proliferation when stimulated with designed peptides. We also detected cellular and humoral immunity activation with IFN-y and antibody production. The data obtained in this study support further development of this immunogen and testing in cats.

## 1. Introduction

The *feline immunodeficiency virus* (FIV) is a retrovirus with global impact and distribution, affecting both domestic and wild felids. FIV infections can cause severe and progressive immunosuppression with the appearance of secondary infections that can lead to death [1]. Genetic studies have allowed the classification of this virus into seven subtypes (A, B, C, D, E, F and U-NZenV) [2]: subtype A is found mainly in Australia, the United States and Europe; subtype B, in Brazil, in Europe, Japan and the United States; subtype C in Canada, Europe, Taiwan and Vietnam; subtype D was isolated in Japan and Vietnam; subtype E was found in Argentina; subtype F in Portugal; and subtype U-NZenV in New Zealand [3,4,5], with a world prevalence rate varying from 2.5 to 31.3% depending on the geographic region and the chosen diagnostic method [6]. Since it was discovered in 1986, some vaccines have been developed and tested but with limited protection against infection. In only 2002, a vaccine approved by USDA [7], named *Fel-O-Vax FIV* (Fort Dodge Animal Health) that uses the entire chemically inactivated virus providing protection against subtypes A and D, started to be used in several countries [8]. Among the difficulties in developing an efficient vaccine against other subtypes is the biology of the virus itself. This is because, although the viral particle has immunogenic epitopes capable of inducing the production of neutralizing antibodies, they also have highly variable epitopes with a high mutation rate that can favor immune escape mechanisms. The highly changeable FIV envelope surface protein has five hypervariable regions (V1–V5), of which the V1, V2 and V3 regions are involved in the entry of the virus into the host cell and as the regions responsible for binding to the CXCR4 coreceptor on the cell surface [9,10,11]. Even if the hypervariable regions V1–V3 can mutate through selective pressure, there is a limitation to the range of possible mutations, as deleterious modifications can impede virus binding to the host cell [9]. Perhaps the success of an FIV vaccine or its failure lies in the way the immune response is orchestrated. The first vaccine construction attempts focused on humoral immune responses, with the production of specific antibodies. Nonetheless, this approach has proven to be not thoroughly efficient for FIV, as vaccinated cats were shown to be consistently more susceptible to the enhancement of the FIV infection than unvaccinated cats, and this was observed in other lentivirus vaccine candidates as well, including HIV and EIAV [12,13]. These data have made it clear that the generation of immunity by only producing antibodies is not enough to inhibit virus replication. Several studies suggest that the development of cellular immunity—mainly cytotoxic T cell responses (CTL)—are essential to control virus replication in the acute phase, shortly after infection [14]. Classic vaccine approaches such as subunit or inactivated vaccines may not be the best way to generate concomitant cellular and humoral responses against FIV. To induce strong cellular and humoral responses, special antigen delivery systems may be required to target proteins of interest to the endogenous antigen processing routes, complex I and II (MHC-I and MHC-II), via major histocompatibility. The technology of recombinant viral vectors is a viable alternative to contemplate such a requirement. Viral vectors are capable of inducing CTL responses against the expressed proteins of interest [12]. The use of recombinant vectors as a vaccination strategy has numerous advantages over traditional technologies, such as: high levels of recombinant antigen production within the cells of the immunized host; the potential adjuvant effect by the proteins of the vector; lyophilization capacity, allowing for storage without specialized refrigeration equipment; the possibility of direct infection of antigen-presenting cells, thus avoiding the previous stages of presentation; and the ability to widely induce not only humoral but also cellular responses [15,16]. Thus, the objective of this work was to develop and evaluate a vaccine prototype against FIV subtype B using the recombinant modified vaccinia Ankara (MVA) virus as a vaccine vector expressing the variable region V1–V3 of the FIV-B envelope. For this trial, we have chosen to use the mouse model due to the lower costs, shorter breading times and easier housing and handling compared to tests in cats. Thus, the model works well preclinically at the phase of immunogenicity testing.

## 2. Materials and Methods

### 2.1. Generation and Production of Recombinant MVA

Our recombinant MVA was engineered to carry the modified coding sequence of the variable region V1–V3 of the FIV, clade B, envelope protein. This gene sequence was amplified from the FIV cDNA collection from a previous work [17] using the primers forward FIV-SmaF (5′-CCTCCCGGGATGTTTAGGGTACCAGGCATTACGTCAC-3′) and reverse FIV-PstR (5′-GGCTGCAGTTACTATTTGTCATCATCATCTTTATAATCTACCGGTTTGGCTCCTGTGAC-3′) designed to insert the SmaI restriction site in the 5′ region and to insert the FLAG peptide and the PstI restriction site in the 3′ region, allowing subcloning in the transfer plasmid pLW44. 

The generated amplicon was inserted into the pGEM-T Easy Vector plasmid (Promega, Madison, WI, USA) according to the instructions provided by the manufacturer and used to transform chemically competent bacteria *Escherichia coli* XL10-Gold^®^. Positive colonies were screened using PCR, and the plasmids were extracted using miniprep (GeneJET Plasmid Miniprep Kit—Thermo Fisher, Waltham, MA, USA) according to the instructions provided by the manufacturer and then sequenced using the Sanger method [18] in Mega Base 1000 (GE HEALTHCARE, Chicago, IL, USA) with the DYEnamic™ ET Dye Terminator kit. The obtained consensus sequence was compared to other subtype B sequences deposited in the “GeneBank” database (http://www.ncbi.nlm.nih.gov/genbank/ accessed on 7 January 2018). After nucleotide sequencing confirmation, the genes of interest (modified *V1–V3* region) were removed from pGEM-T and subcloned into pLW44; this vector has 2 MVA genome flanks, the promoters p11 and mH5 and the green fluorescent protein (GFP) coding gene as a reporter. The construction of the recombinant viruses was based on homologous recombination, in which chicken embryo fibroblast cells (CEF) were transfected with the transfer plasmid pLW44/FIV-B and then infected with wild type MVA (WT-MVA). After this, the recombinant MVA viruses (MVA/FIV-B) were selected through successive plaque purification assays and, after 10 rounds of selection, the recombinant viruses were amplified, purified and titrated as described [19]. 

### 2.2. Western Blot

Monolayers of baby hamster kidneys cells, (BHK-21)—ATCC CCL-10^TM^, were inoculated with multiplicity of infection (MOI) = 5 of recombinant MVA/FIV-B virus. After 48 h, cell monolayers were collected, and whole cell proteins were extracted [20]; uninfected BHK-21 cells were used as mock controls, and a recombinant FIV p24 protein was used as a positive control. A sera pool of five FIV^+^ animals was used to perform western blot, as previously described [21].

### 2.3. Shelf Life

To test the biological viability of the vaccine product, the virus was titrated in three stages for comparison purposes. The first consisted of the titration of the virus stored at −80 °C. The second consisted of the titration of the virus after lyophilization, and the last step was the titration of the lyophilized virus after 3 months of storage in refrigerator at 4–8 °C. The titration of these virus samples steps was performed by the infection of BHK-21 cell monolayer with the recombinant virus, and after 48 h of incubation, the clones were counted under fluorescence microscopy.

### 2.4. Immunization Protocol

In this study, we used 7 week-old male C57/BL, obtained from the central vivarium of the Institute of Biological Sciences at Federal University of Minas Gerais (UFMG). The procedures used here were approved by the Animal Experimentation Ethics Committee (CEUA) of UFMG, under protocol #3355/2013. Mice were divided in three groups (n = 6) and received one dose of 40 µL of intramuscular inoculum with sterile 10 mM Tris-HCl or 10^−6^ pfu/mL of recombinant virus MVA/FIV-B or WT-MVA virus, both diluted in 10 mM Tris-HCl. After 28 days, all animals received a booster dose equal to the first dose, and 14 days after booster dose, the animals were euthanized. Spleens and blood were harvested, and serum was separated from whole blood by centrifugation for 10 min at 600× *g* and stored at −20 °C until use.

### 2.5. Synthetic Peptide Design

In order to carry out the intracytoplasmic cytokine assays (ICs), ELISA and splenocytes proliferation assays (SPA), seven peptides from the *V1–V3* region of the FIV-B envelope were synthesized. In silico prediction of immunogenic epitopes for specific TCD8^+^ cells alleles of MHC class I molecules H2-Kb and H2-Db was performed using the IEDB platform (http://tools.immuneepitope.org/analyze/htmL/mhc_binding.htmL Accessed on 4 February 2018). The synthetic peptides (GeneOne—São Paulo, Brazil) were diluted in dimethyl sulfoxide (DMSO) to a final concentration of 10 mg/mL and stored at −80 °C until use. 

### 2.6. Intracytoplasmic Cytokines

To confirm the immunogenicity of the in silico predicted epitopes, an intracytoplasmic cytokine labeling assay was performed. Spleen cells were isolated as previously described [22]. Mice immunized with the recombinant MVA/FIV-B virus were stimulated with the peptides to determinate the best cell responses. After restimulating with each peptide diluted at 10^−5^, the TCD8^+^ IFN-γ production was evaluated as previously described [23], and sample analysis was performed on a FACSCalibur cytometer (Becton Dickinson, Franklin Lakes, NJ, USA).

### 2.7. Enzyme-Linked Immunosorbent Assay, ELISA

For peptide-based ELISA, synthetic peptides were diluted in 50 µL of milli-Q H_2_O to a final amount 2 µg per well in 96 wells ELISA plates. Pure H_2_O was used as a negative control. The plate was incubated at 37 °C overnight, then blocked with 150 µL of 2.5% casein in PBS 1X for 1 h at 37 °C. Next, we performed three wash cycles with washing solution (1x PBS and 0.05% Tween 20). Sera samples were diluted (1:100) in incubation buffer (1.25% casein in PBS 1X) and incubated for 1 h at 37 °C, followed by three wash cycles in washing solution. Then, 100 µL of conjugate (1:40,000 goat anti-mouse IgG, human ads-HRP) were added and incubated for 60 min at 37 °C. After incubation, the plate was washed three times, and 100 µL of the revealing solution was added (10 mL of citrate buffer, 10 mg of OPD and 10 mg of hydrogen peroxide (H_2_O_2_)) and incubated for 25 min at room temperature in the dark. At the end, the reaction was stopped with 25 µL of H_2_SO_4_ at final concentration of 0.92 mol/L, and results were detected in a spectrophotometer at 492 nm wavelength setting.

### 2.8. Splenocytes Proliferation Assay (SPA)

The proliferative splenocytes were tracked by the intracellular dye carboxyfluorescein diacetate succinimidyl ester (CFSE) (CFSE cell proliferation kit, Invitrogen, Waltham MA, USA, Molecular Probes), diluted in DMSO according to the manufacturer instructions. The assay was performed as previously described [24]. Splenocytes were separated by the stimulus groups (peptide 2, SMVYLLIGYL) at a dilution of 10^−5^, mock (culture medium RPMI) or positive control (concavalin A (ConA) (4 μg/mL, Sigma-Aldrich, St. Louis, MO, USA)).

### 2.9. Statistical Analysis

All variables were tested for normality of distribution by the Shapiro–Wilk test. Analysis of variance (ANOVA), multiple comparison (Kruskal–Wallis test) and unpaired *t*-test were conducted to evaluate the data. The significance level for the results was set at *p* < 0.05. Statistical analyses were performed using GraphPad Prism 6 ^®^ Software (Dotmatics, Boston, MA, USA). 

## 3. Results

### 3.1. Generation and In Vivo Characterization of MVA Vectors Encoding the V1–V3 Variable Region of the FIV Envelope Protein

**Figure 1:**
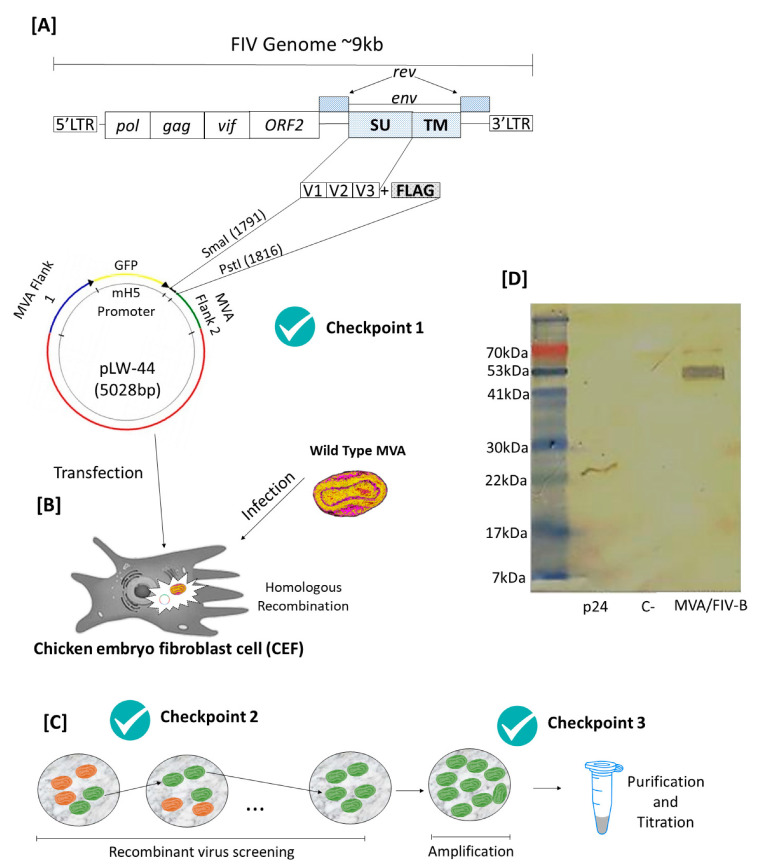
The construction process of the recombinant virus MVA/FIV-B. (**A**) Obtaining the V1–V3 sequence and inserting the FLAG portion and restriction sites for further cloning in plasmid pW-44. (**B**) Homologous recombination in the chicken embryo cell (CEF) by transfection with plasmid pLW-44/FIV-V1–V3 and infection with wild MVA. (**C**) Selection process of recombinant clones, followed by amplification, purification and titration. (**D**) Western blot using FIV^+^ cat sera as pool. P24 represents the viral capsid protein used to diagnose FIV. MVA/FIV represents the total protein extraction of BHK-21 cells infected with MVA/FIV-B, and C^-^ represents total proteins extracted from uninfected BHk-21 cells.

First, specific primers were designed for the V1–V3 region of the FIV envelope gene, but at the same time, these primers were responsible for inserting, by PCR, restriction sites and the FLAG peptide coding into the amplicon, generating a 1389 bp product. The PCR product was used in the construction of the transfer plasmid (Figure 1A). To ensure the correct construction of the plasmid pLW-44, PCR, enzymatic digestion and sequencing were performed (Checkpoint 1). Chicken embryo cells (CEFs) were transfected with pLW-44 containing the gene of interest and infected with the WT-MVA virus to promote homologous recombination (Figure 1B). Due to the fact that pLW44 inserts the GFP gene, the recombinant viruses were selected by the presence of fluorescence (Figure 1C). In addition, PCR and RT-PCR were performed to assess the presence of the insert and the production of mRNA (Checkpoints 2 and 3). At the end of the amplification and screening process, western blotting was performed to detect the presence of the FLAG peptide, linked to the recombinant protein, named FLAG-ENV/1-3, with 55 kDa of molecular weight, produced by the recombinant MVA/FIV-B virus. Next, we determined the response of the sera from cats naturally infected with FIV to the recombinant protein. Using the sera pool from five FIV-positive animals, we performed a western blot where our data showed the antibody recognition from naturally infected cats to the recombinant proteins as well to the viral capsid protein P24 used as a positive control (Figure 1D). 

#### Storage Stability

The viability of the recombinant MVA/FIV-B virus was verified after lyophilization, followed by storage for three months at a temperature of 4–8 °C. This is a basic assessment for any vaccine product that is intended to be clinically evaluated and marketed in the future. The data shown in Table 1 indicate that, after the storage time, there was no drop in viral titers, and the same results were verified after three months in refrigerator storage.

### 3.2. Recombinant MVA-FIV Induces Immunological Responses in Mice

#### 3.2.1. Interferon-γ Production in Response to Synthetic Peptides Stimuli

**Figure 2:**
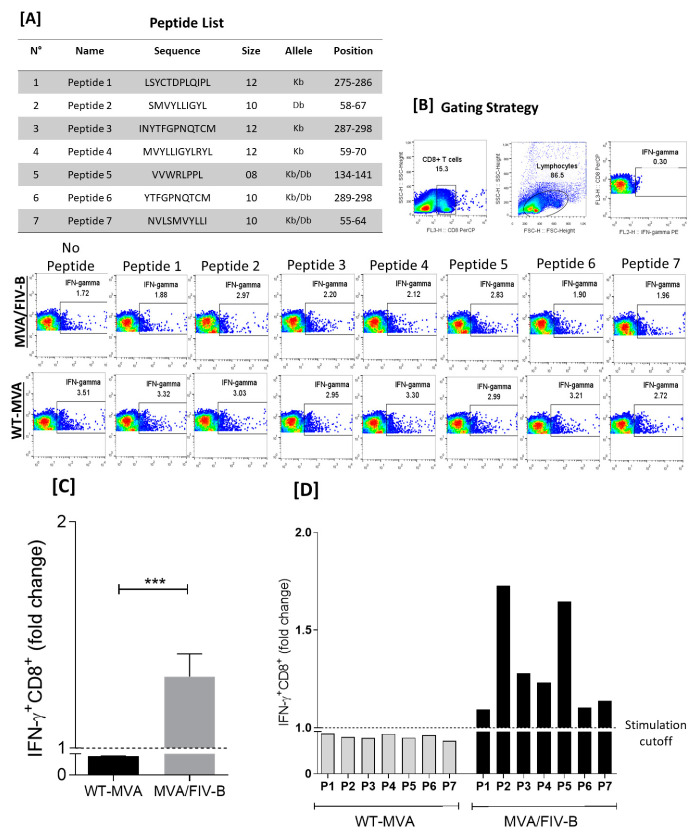
Intracytoplasmic cytokine. (**A**) List of epitopes mapped in the FIV env gene and the gatting strategy used to quantify the production of INF-γ produced by TCD8^+^ cells. (**B**) Gating strategy and the peptide stimulus in immunized mice with WT-MVA and recombinant MVA/FIV-B. (**C**) Fold change graph of the average stimulation of synthetic peptides in the group of animals immunized with MVA/FIV-B and WT-MVA (*p* > 0.001). (**D**) Fold change graph of the individualized stimulation of the 7 synthetic peptides, showing that peptide 2 and peptide 5 present a better stimulation profile, inducing a greater production of INF-γ. Gray bars are form WT-MVA and black bars are form MVA/FIV-B. *** *p* < 0.001.

To verify the specific IFN-γ response of TCD8^+^ cells in the murine model, we first identified and synthesized seven putative T cell epitopes present in the *V1–V3* region of the FIV *env* gene (Figure 2A). The animals were immunized using the prime/boost protocol (14 days apart) with recombinant MVA/FIV-B virus or WT-MVA. On the 14th day after the last dose, the splenocytes were collected and restimulated with the synthetic peptides at a final dilution of 10^−5^ to assess which epitope had the best potential to induce IFN-γ production in TCD8^+^ cells (Figure 2B). In summary, the seven evaluated peptides showed statistically superior IFN-γ production among animals immunized with the recombinant virus compared to WT-MVA after stimulation (*p* = 0.001) (Figure 2C). Analyzing the peptides performance separately, we observed a better stimulation induced by peptide 2 (SMVYLLIGYL) and peptide 5 (VVWRLPPL) compared to the others. These results led us to use these in the next analyses.

#### 3.2.2. Antibody Responses to Recombinant Protein and Synthetic Peptides

**Figure 3:**
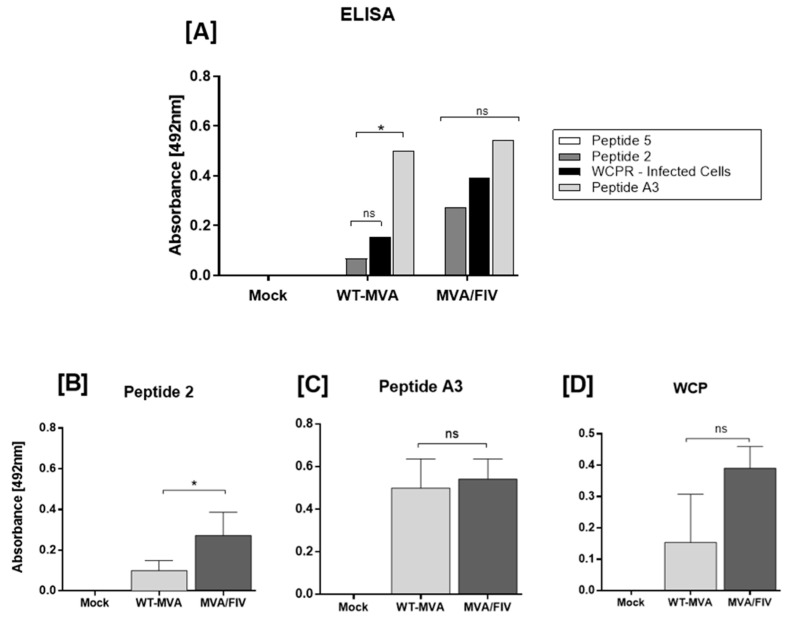
Peptide ELISA. (**A**) Overview of the serum reaction of the mock, WT-MVA and MVA/FIV-B groups to the presented substrates, peptide 2, peptide 5, WCP and peptide A3. (**B**) Response to peptide 2 where a significant difference is observed between WT-MVA and MVA/FIV-B, while Mock remained nonreactive. (**C**) Response to control, peptide A3. It is observed that animals immunized with viruses (WT-MVA or MVA/FIV-B) reacted in a very similar way, while the mock remained nonreactive. (**D**) Response to the WCP substrate composed of protein extract from BHK-21 cells infected with MVA/FIV-B. It is observed that the WT-MVA group showed a low reactivity compared with the MVA/FIV-B group, although this difference is not statistically specific. Mock group remained nonreactive. * *p* < 0.05.

This assay was performed with the serum of the three study groups (MVA/FIV-B, WT-MVA and mock) obtained 14 days after the last immunization. As substrates, we used the A3 peptide from the MVA virus framework, whole cell protein (WCP) extracted from BHK-21 monolayer infected with the MVA/FIV-B virus, and peptides 2 and 5, which presented a better induction profile regarding the production of IFN-γ by TCD8^+^ cells. The ELISA showed that the mock group was negative for all tested substrates (Figure 3A). Peptide 5 did not show reactivity in any group. When analyzing the substrates individually, peptide 2 showed greater reactivity in the MVA/FIV-B group than in the WT-MVA group, with a significant difference (*p* < 0.05), suggesting that the response found in the WT-MVA group is nonspecific (Figure 3B). The A3 peptide, being part of the MVA framework, was used as a positive control in this assay. It was possible to notice in Figure 3C that it showed reactivity in both groups immunized with viruses, showing no significant difference between them, corroborating that the animals were properly immunized. Even though substrate WCP showed low reactivity in the WT-MVA group and high reactivity in the MVA/FIV-B, there is no statistical difference between them, suggesting that both groups are reacting to WCP. While the group WT-MVA is reacting to the proteins of the vector itself, the group MVA/FIV-B is reacting to the vector proteins and the recombinant FIV peptides (Figure 3D).

#### 3.2.3. Cell Proliferation Responses to Synthetic Peptide

**Figure 4:**
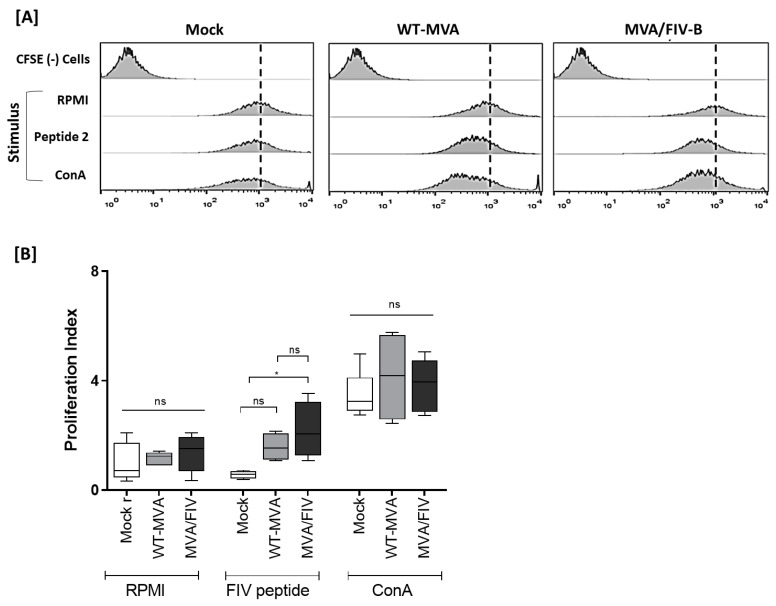
CFSE of splenocytes of the immunized and nonimmunized animals. (**A**) The splenocytes of animals immunized with MVA/FIV, WT-MVA and mock-infected were subjected to different stimuli and compared to unlabeled cells. Shifts are observed at the left side of each graph. (**B**) Proliferation index where it is possible to observe that stimulation by RPMI did not present any statistical difference and maintained a lower proliferation index compared to other stimuli. Peptide stimulation did not significantly differentiate the WT-MVA group with MVA/FIV-B and mock, while the MVA/FIV-B showed a significant difference (* *p* < 0.05) when compared to mock-inoculated animals, showing a higher proliferation index. Concavalin A (ConA) stimulus was used as a positive control.

CFSE dye was used to assess the proliferative response of splenocytes. After 14 days from the last immunization, the splenocytes of the animals were collected, marked with CFSE, stimulated with ConA, RPMI or peptide2, and cultured for 5 days. The proliferation rate was analyzed using flow cytometry detecting the displacement of the cell population compared with the control of cells not marked with CFSE. In this assay, three stimuli (RPMI, ConA and Peptide 2) were used to access the proliferation of the groups of immunized and nonimmunized animals. The RPMI stimulus used as a negative control did not significantly stimulate the proliferation of any of the immunized and nonimmunized groups. In contrast, ConA, used as a positive proliferation control for inducing nonspecific cell proliferation, had the highest proliferation rates in all groups evaluated, with no significant difference between them. The peptide 2 stimulus did not significantly increase proliferation in the mock and WT-MVA groups, with no significant difference between them; however, this stimulus was able to increase cell proliferation in the recombinant MVA/FIV-B group, with a significant difference (*p* < 0.05) compared to the mock group (Figure 4).

## 4. Discussion

Since its discovery in 1986, numerous attempts to develop an effective vaccine for FIV have been made [5,12,25,26]. Currently, of the seven viral subtypes circulating worldwide, we have vaccines available for subtypes A and D only. It is important to highlight that subtypes A, B and C are the most widespread [4,5,14]. For this reason, this work aimed to develop a vaccine prototype against FIV subtype B, which is the predominant subtype found in many regions, including Brazil. Many previous studies pointed out that the best strategy for developing an FIV or HIV vaccine would be an immunogen that activates both cellular and humoral immune responses [2,12]. The strategy of this study was based on the recombinant vector technology, using the MVA to carry in its genome a modified gene coding the variable regions *V1–V3* of the FIV subtype B envelope protein, known to be responsible for virus entry into the host cell [9]. 

After the construction of the vector MVA/FIV-B, the shelf life was evaluated upon lyophilization followed by a period of three months in refrigerator temperature without stabilizers. The results show that MVA/FIV-B did not decrease the viral titers over time, corroborating previous works that have shown the stability of MVA upon storage under different conditions and for periods longer than one year [27,28]. This is a positive quality that favors the distribution of this vaccine, giving a commercial advantage to the product.

IFN-γ is a known promoter of antiviral responses, increasing antigen processing, inflammation and macrophage differentiation and is mainly produced by T lymphocytes and natural killer cells [29]. To assess cell responses induced by our vaccine vector, we synthetized seven putative T cell epitope peptides found within the V1–V3 hypervariable region of FIV envelope protein. The cellular immune responses were determined by the amount of IFN-γ produced by the splenocytes of immunized mice. As these epitopes are not part of the MVA structure, they generated a specific and greater response in animals inoculated with recombinant MVA/FIV-B virus. The best peptide performances were obtained for peptides 2 (SMVYLLIGYL) and 5 (VVWRLPPL), out of the seven tested. Furthermore, the proliferative responses of LTCD8^+^ induced by vaccination with recombinant MVA/FIV-B was measured by a CFSE dye assay using peptide 2 as the specific stimulus and ConA as the nonspecific cell proliferator [30]. The immunized animals with the vaccine vector showed the highest proliferation index with peptide 2 compared with other groups. These findings suggest a high level of specific response and the ability of the recombinant vector to activate cells leading to cell proliferation after vaccination. This is a crucial factor because studies have reported the importance of T cell immunity against FIV [7,31,32].

To evaluate some aspects of the humoral responses, the ability of FIV-positive cat serum to identify the recombinant protein (FLAG-ENV/1–3) produced by the MVA/FIV-B virus in infected cells was determined by western blot assays. The data reported the ability of the vaccine to produce a heterologous protein capable of being recognized by the serum of animals naturally infected with FIV. These results corroborate that MVA is a vector capable of being engineered to produce heterologous proteins with the correct folding in mammal systems, which was extensively studied and reviewed elsewhere [33,34,35,36].

The ELISA assay suggests the specific immune response generated by the vaccine. In fact, the use of peptide 2 as a solid phase showed a statistically significant difference between the immunized groups and a higher index in recombinant MVA/FIV-B-immunized animals. A possible explanation for the response of the protein extract from infected cells with MVA/FIV-B, observed in animals inoculated with WT-MVA, is that the protein extract used in the study has native MVA proteins (vector proteins) and FIV envelope V1–V3 proteins (heterologous proteins). This suggests a vector recognition by the serum of animals inoculated with WT-MVA, while the group inoculated with recombinant MVA/FIV-B shows both, a recognition to vector proteins and specific heterologous proteins which can be indirectly measured by the ratio between the groups. The response to the MVA vector was reported in other studies [37].

Although there is a commercially available vaccine against FIV subtype A and D (Fel-O-Vax FIV; Fort Doge) licensed since 2002 in EUA and some other countries [38], this vaccine is controversial, and the fact that not all the countries have implemented it in routines is due to two main reasons: first, the efficacy of the vaccine is debatable, as reviewed by [39]. Second, the vaccine contains FIV-inactivated particles in its composition, and this can lead to a diagnostic dilemma because vaccinated cats can test positive for FIV in tests that detect antibodies against the viral proteins P15, P24, gp40 such as SNAP Combo (IDEXX Laboratories, Westbrook, Maine, USA), Witness (Zoetis laboratory, Louisville, KY, USA) and Antigen Rapid (BioNote laboratory, Minneapolis, Minnesota, USA). False positive results are an important issue because they can lead to unnecessary euthanasia [2]. 

The main purpose of this study was to develop a new recombinant MVA-vectored vaccine against the FIV subtype B to particularly increase cell responses. We used the mouse model to assess humoral and cellular responses after vaccination. Despite advantages such as lower costs and easier housing, mice are not susceptible to FIV infection, precluding challenge experiments. It is known that subunit and/or inactivated vaccines (currently available) are poor inducers of cellular responses, and such responses are generally known to be essential to control the replication of retroviruses. Therefore, any increase in cellular responses can be significant. However, immunogenicity of MVA/FIV-B has to be improved. For that, new dose-escalating studies with proper animal models such as felines are still required to evaluate real immunogenicity and improve immune responses induced by this new vaccine prototype. 

## 5. Conclusions

We have demonstrated that the recombinant MVA virus engineered to carry the V1, V2 and V3 hypervariable regions of the FIV envelope protein as a prototype vaccine for a FIV subtype B was able to activate cellular and humoral immune responses evaluated in the murine model at a preclinical stage. Among the advantages of this vaccine technology are the maintaining of biological characteristics after lyophilization and not generating false positive results in vaccinated animals when tested by the most frequent test kits. Our results support the clinical assessment of the constructed immunogen. 

## Figures and Tables

**Table 1:** Title of lyophilized and nonlyophilized MVA/FIV-B virus.

Storage Time	Viral Title (UFP/mL)
MVA/FIV-B stored at −80 °C	7.3 × 10^9^
MVA/FIV-B after lyophilization	7.2 × 10^9^
MVA/FIV-B lyophilized and after 3 months at 4–8 °C	2.7 × 10^9^

## Data Availability

Not applicable.
